# Comparison of Functional Outcomes Between Short and Long Cephalomedullary Nails in Intertrochanteric Femur Fractures in Elderly Patients

**DOI:** 10.7759/cureus.89818

**Published:** 2025-08-11

**Authors:** Mani Bagga, Sanjeev Mahawar, Gautam Chatterji, Rajat Nagar, Rishi Parashar

**Affiliations:** 1 Department of Orthopaedics, People's College of Medical Sciences & Research Centre, Bhopal, IND

**Keywords:** cephalomedullary nails, elderly patients, harris hip score, intertrochanteric femur fractures, proximal femur nail, visual analogue scale

## Abstract

Introduction: Cephalomedullary nails (CMNs) are the most commonly used surgical implants for the treatment of intertrochanteric fractures in the geriatric population. However, there remains ongoing debate regarding the long-term benefits and complication risks associated with the length of CMNs, short (<250 mm) versus long (>250 mm), in managing these fractures. This study aims to compare the functional outcomes between short and long CMNs using the Harris Hip Score (HHS) and assess the complication rates associated with each type.

Method: The study included 50 patients aged 60-85 years who underwent cephalomedullary femoral nailing, either short or long, for Association for Osteosynthesis/Orthopaedic Trauma Association (AO/OTA) type 31A1 and 31A2 trochanteric femur fractures. Data collected included duration of hospitalization, operative time, intraoperative blood loss, time to radiological union, time to weight-bearing, postoperative pain assessed using the Visual Analogue Scale (VAS), and complications such as anterior thigh pain and screw cutout or backout. Functional outcomes were evaluated using the HHS. Follow-ups were conducted at 5 days, 14 days, 30 days, 3 months, 6 months, and 8 months postoperatively.

Results: A total of 25 patients were treated with short and long CMNs. The short nail group showed significantly lower intraoperative blood loss (94.84 ± 11.1 mL vs. 117.24 ± 25.81 mL, p = 0.01) and shorter operative time (51.84 ± 4.37 min vs. 56.40 ± 2.74 min, p = 0.01). No significant differences were observed in hospital stay, time to weight-bearing, or radiological union. At eight months, the VAS score was significantly better in the short nail group (p = 0.002). HHS scores were comparable in the early postoperative period but significantly favored the short nail group at three months (p = 0.00), six months (p = 0.002), and eight months (p = 0.02). Screw backout occurred in one patient (4%) in the long nail group and two patients (8%) in the short nail group (p = 0.551), while anterior thigh pain was reported in five patients (20%) in the short nail group and one patient (4%) in the long nail group (p = 0.081); however, these complications were not statistically significant.

Conclusion: This study contributes to the existing body of evidence by demonstrating that both short and long CMNs are viable treatment options for trochanteric fractures. However, our findings indicate that short CMNs may offer certain advantages, including reduced intraoperative blood loss, shorter operative time, and improved functional outcomes as assessed by the HHS.

## Introduction

Proximal femoral fractures are among the most common fractures observed in the elderly population worldwide, with a global incidence exceeding 182.5 per 100,000 person-years. As the global population ages and life expectancy continues to rise, the burden these fractures impose on healthcare systems is expected to increase significantly [1]. Hospital-based studies indicate that hip fractures are prevalent in India as well. A recent study from North India reported an annual incidence rate of 163 per 100,000 in women and 121 per 100,000 in men over the age of 55. Interestingly, hip fractures appear to occur at a younger age in the Indian population compared to Western counterparts, with the peak incidence occurring in individuals in their 60s [2]. Intertrochanteric fractures are a significant source of morbidity, mortality, and functional decline in the elderly [3]. These fractures are defined as extracapsular breaks in the proximal femur occurring between the greater and lesser trochanters [4]. The primary objective of surgical intervention in such cases is to restore mobility [5]. Early mobilization has been shown to reduce complications associated with prolonged bed rest, such as pneumonia, deep vein thrombosis, pulmonary embolism, and even mortality. Consequently, early postoperative mobilization has become a standard practice [6]. Over the past decade, cephalomedullary nailing has emerged as the preferred surgical technique for managing trochanteric fractures. Its key advantages include the facilitation of early mobilization and accelerated rehabilitation [5]. Cephalomedullary nails (CMNs) also offer superior biomechanical properties compared to plates, dynamic hip screws, and external fixation devices in the management of unstable trochanteric fractures. These nails provide enhanced stability and withstand higher mechanical loads [7]. Despite these benefits, debate persists regarding the long-term efficacy and safety of short (<250 mm) versus long (>250 mm) CMNs [8,9]. Short nails, while beneficial in terms of reduced operative time and blood loss, have historically been associated with a higher risk of peri-implant fractures due to stress risers at the distal tip. However, advancements in nail design have addressed many of these concerns [10]. Current literature supports the use of both short and long CMNs for AO type 31A1 and 31A2 intertrochanteric femur fractures. Comparing short versus long CMNs in the management of AO type 31A1 and 31A2 fractures is clinically important because it involves a trade-off between surgical ease and stability. Short CMNs offer advantages such as shorter operative time, reduced intraoperative blood loss, and easier insertion, which can be beneficial in elderly patients with multiple comorbidities. Conversely, long CMNs provide increased biomechanical stability by spanning the entire femoral shaft, potentially reducing the risk of peri-implant and secondary femur fractures, especially in osteoporotic bone. This balance between minimizing surgical trauma and maximizing implant stability is crucial for optimizing patient outcomes and guiding appropriate treatment strategies.

## Materials and methods

Study setting, design, and study participants

This prospective observational study was conducted in the Department of Orthopaedics at People's Hospital, Bhanpur, affiliated with the People's College of Medical Sciences & Research Centre, a tertiary care center. A total of 50 elderly patients with intertrochanteric femur fractures were included and treated with cephalomedullary femoral nailing between November 2022 and January 2024. All surgeries were performed after obtaining approval from the Institutional Ethics Committee (approval number IEC-2022/79) and written informed consent from the patients.

The inclusion criteria consisted of closed fractures classified as AO type 31A1 and 31A2 in patients aged between 60 and 85 years. Patients were excluded if they were younger than 60 or older than 85 years, or if they had AO type 31A3 fractures, open fractures, or pathological fractures.

Operative procedure

Following ethical clearance and informed consent, patients were enrolled and underwent surgery using a minimally invasive technique for intramedullary nail insertion, employing either a short or long CMN. The choice of nail length was made exclusively by the primary operating surgeon, without any input from the investigators or co-authors.

The CMNs used in this study had standardized specifications. The short nail measured 25 cm in length, while the long nails were available in lengths of 36, 38, 40, and 42 cm. All nails had a proximal diameter of 15 mm and a proximal angulation of 6 degrees. The distal diameters included 9, 10, 11, and 12 mm. The lag screw had a diameter of 8 mm, the de-rotation screw measured 6.2 mm, and the distal locking bolt was 4.9 mm in diameter.

The surgical protocol for patients treated with short or long CMN for AO type 31A1 and 31A2 proximal femoral fractures involved positioning the patient supine on a fracture table, facilitating adduction of the affected leg to allow proper nail insertion exclusively at the piriformis fossa under fluoroscopic guidance. Closed reduction was performed under fluoroscopy to achieve anatomical alignment, followed by insertion of a guide wire centrally in the medullary canal. Sequential reaming of the femoral canal was done as needed based on nail size, after which the appropriately sized CMN was inserted until the proximal tip was flush with the piriformis entry site. A lag screw and a de-rotation screw were then placed through the nail into the femoral head and neck to maximize fixation stability and rotational control, positioned centrally or slightly inferiorly as indicated by the fracture pattern. Distal locking screws were inserted to secure the implant and maintain alignment and length. Intraoperative fluoroscopy ensured the correct positioning of both the implant and fracture reduction. Prophylactic antibiotics were administered perioperatively, and careful surgical technique was emphasized to avoid complications such as screw cutout. 

Postoperative protocol

The postoperative protocol involved initiating quadriceps and hip range-of-motion exercises, limb elevation, and thromboprophylaxis with low-molecular-weight heparin in the early postoperative period. Partial weight-bearing with walking aids, such as crutches, was permitted at six weeks postoperatively, with patients being maintained non-weight-bearing prior to this time. Full weight-bearing was permitted at 10 weeks, once clinical and radiological signs of healing were evident, and patients subsequently advanced to independent ambulation as tolerated.

Postoperative assessment

Postoperative evaluation included the Harris Hip Score (HHS) for functional outcomes and the Visual Analogue Scale (VAS) for pain assessment at 14 days, 30 days, 3 months, 6 months, and 8 months following surgery. The HHS was used as per the original description by Harris [11]; no modifications were made. Additional data collected included duration of hospitalization, mean operative time, mean blood loss, time to radiological union, time to full weight-bearing, and complications such as anterior thigh pain and screw cutout or backout.

Statistical analysis

Data were compiled using Microsoft Excel (Microsoft Corp., Redmond, WA, US) and analyzed using IBM SPSS Statistics for Windows, Version 20.0 (Released 2011; IBM Corp., Armonk, NY, US). Categorical data were expressed as frequencies and percentages, while continuous data were presented as means and standard deviations. The chi-square test was used for categorical variables, and the independent t-test was applied to compare continuous variables between the two groups. A p-value of less than 0.05 was considered statistically significant.

## Results

A total of 50 subjects participated in the study, with 25 assigned to the long CMN group and 25 to the short CMN group. Baseline characteristics of the participants are summarized in Table 1. 

**Table 1:** Preoperative patient characteristics in short and long CMN groups AO/OTA: Association for Osteosynthesis/Orthopaedic Trauma Association, CMN: cephalomedullary nail.

Patient variables	Long CMN	Short CMN
Age (in years)		
60-70	17 (68%)	14 (56%)
71-80	7 (28%)	7 (28%)
81-85	1 (4%)	4 (16%)
Gender		
Female	13 (52%)	7 (28%)
Male	12 (48%)	18 (72%)
Side of injury		
Left	10 (40%)	11 (44%)
Right	15 (60%)	14 (56%)
Mode of injury		
Fall	14 (56%)	19 (76%)
Road traffic accident	11 (44%)	6 (24%)
AO/OTA classification		
AO 31A1	12 (48%)	11 (44%)
AO 31A2	13 (52%)	14 (56%)

The mean operative time was significantly lower in the short CMN group, averaging 51.84 minutes, compared to 56.60 minutes in the long CMN group (p < 0.01).

Similarly, the mean intraoperative blood loss was significantly reduced in the short CMN group, with an average of 94.84 mL, compared to 117.24 mL in the long CMN group (p < 0.01). These findings are summarized in Table 2.

**Table 2:** Intraoperative assessment of short and long CMN groups Independent t-tests were used to compare long and short CMNs with respect to the intraoperative parameters of mean operative time and mean blood loss. A p-value of less than 0.05 was considered statistically significant. CMN: cephalomedullary nail.

Variable	Long CMN	Short CMN	t-value	p-value
Mean operative time (in minutes)	56.40 ± 2.74	51.84 ± 4.37	4.417	<0.01
Mean blood loss (in mL)	117.24 ± 25.81	94.84 ± 11.11	3.986	<0.01

The functional outcome, assessed using the HHS, consistently favored the short CMN group at postoperative intervals of 30 days, 3 months, 6 months, and 8 months. Details of the HHS outcomes are provided in Table 3.

**Table 3:** Harris Hip Score as an indicator of functional outcome in short and long CMN groups p-values from independent t-tests were used to compare the Harris Hip Scores of patients treated with short and long CMNs at various postoperative time intervals. A p-value of less than 0.05 was considered statistically significant. CMN: cephalomedullary nail.

Postoperative time interval		Mean	Standard deviation	t-value	p-value
5 days	Long CMN	29.68	3.64	0.324	0.747
	Short CMN	29.35	3.64		
14 days	Long CMN	39.14	2.64	-0.481	0.632
	Short CMN	38.77	2.80		
30 days	Long CMN	67.04	5.54	2.12	0.02
	Short CMN	69.92	3.51		
3 months	Long CMN	75.55	3.44	3.60	0.000
	Short CMN	78.52	2.27		
6 months	Long CMN	79.30	2.78	3.18	0.002
	Short CMN	81.82	2.82		
8 months	Long CMN	84.96	4.71	2.28	0.02
	Short CMN	88.17	5.23		

Postoperative pain levels, measured by the VAS, showed no significant difference between the groups at most follow-up points. However, at the 8-month follow-up, the short CMN group demonstrated a significantly lower VAS score. The data are detailed in Table 4.

**Table 4:** Comparison of mean VAS score of study subjects between the two groups at different time intervals p-values from independent t-tests were used to compare VAS scores between patients treated with short and long CMNs at various postoperative time intervals. A p-value of less than 0.05 was considered statistically significant. VAS: Visual Analogue Scale, CMN: cephalomedullary nail.

Postoperative time interval		Mean	Standard deviation	t-value	p-value
5 days	Long CMN	69.60	7.35	0.00	0.459
	Short CMN	71.20	7.81		
14 days	Long CMN	62	7.63	-0.746	0.84
	Short CMN	61.6	6.24		
30 days	Long CMN	50.40	10.20	-0.20	0.553
	Short CMN	52.00	8.66		
3 months	Long CMN	43.2	11.07	-0.598	0.22
	Short CMN	39.6	9.78		
6 months	Long CMN	32.4	13.62	-1.55	0.266
	Short CMN	26.8	11.80		
8 months	Long CMN	28.4	17.48	-3.199	0.002
	Short CMN	18.00	14.43		

There were no statistically significant differences between the groups regarding postoperative complications, including time to radiological union, screw backout, thigh pain, or the ability to bear weight. These findings are summarized in Table 5.

**Table 5:** Complications and postoperative assessment in short and long CMN groups p-values from independent t-tests were used to compare the mean duration of postoperative hospital stay (in days) and the time to radiological union (in weeks) between patients treated with short and long CMNs. Similarly, p-values from chi-square tests were used to compare the incidence of screw cutout or backout, anterior or lateral thigh pain, and the ability to bear weight at various postoperative time intervals between the two groups. A p-value of less than 0.05 was considered statistically significant. CMN: cephalomedullary nail.

Variable	Long CMN	Short CMN	Test used	Test statistic	p-value
Mean duration of postoperative hospital stay (in days)	5.60 ± 0.65	5.76 ± 1.05	Independent t-test	t-value = -0.65	0.521
Time for radiological union (in weeks)	17.16 ± 1.34	17.12 ± 1.42	Independent t-test	t-value = 0.102	0.919
Screw cutout or backout	1 (4%)	2 (8%)	Chi-square test	X^2^ = 0.354	0.551
Anterior or lateral thigh pain	1 (4%)	5 (20%)	Chi-square test	X^2^ = 3.03	0.081
Ability to bear weight					
At 3 months post-op	19 (76%)	21 (84%)	Chi-square test	X^2^ = 0.814	0.366
At 6 months post-op	22 (88%)	24 (96%)	Chi-square test	X^2^ = 0.452	0.501
At 8 months post-op	23 (92%)	24 (96%)	Chi-square test	X^2^ = 0.354	0.551

**Figure 1:**
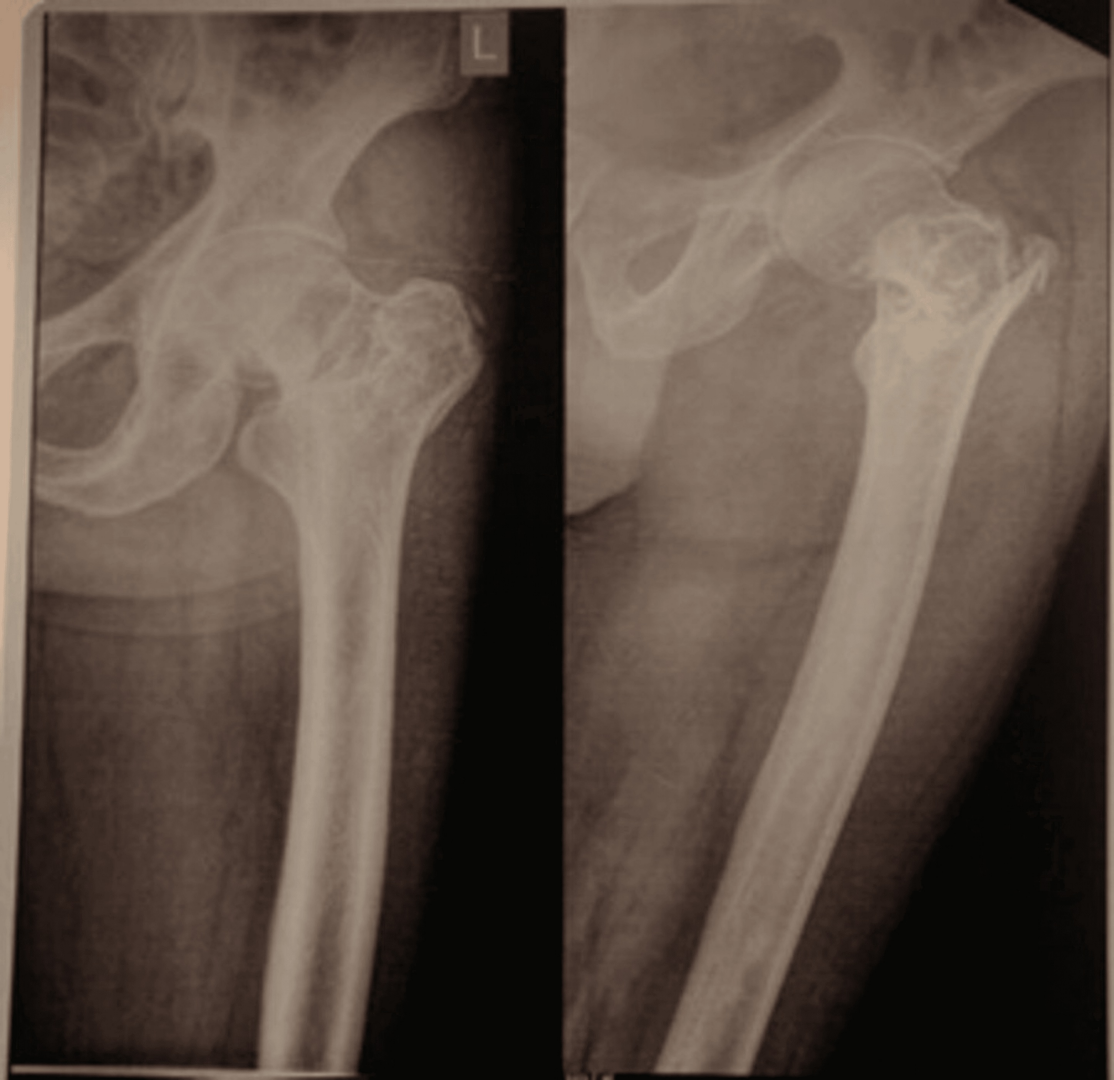
Preoperative radiograph of an adult patient showing left hip intertrochanteric fracture These are anteroposterior (AP) and lateral projections of the preoperative radiograph of the left hip joint and proximal femur of a patient included in the study, showing AO/OTA: Association for Osteosynthesis/Orthopaedic Trauma Association (AO/OTA) type 31A1 intertrochanteric fracture.

**Figure 2:**
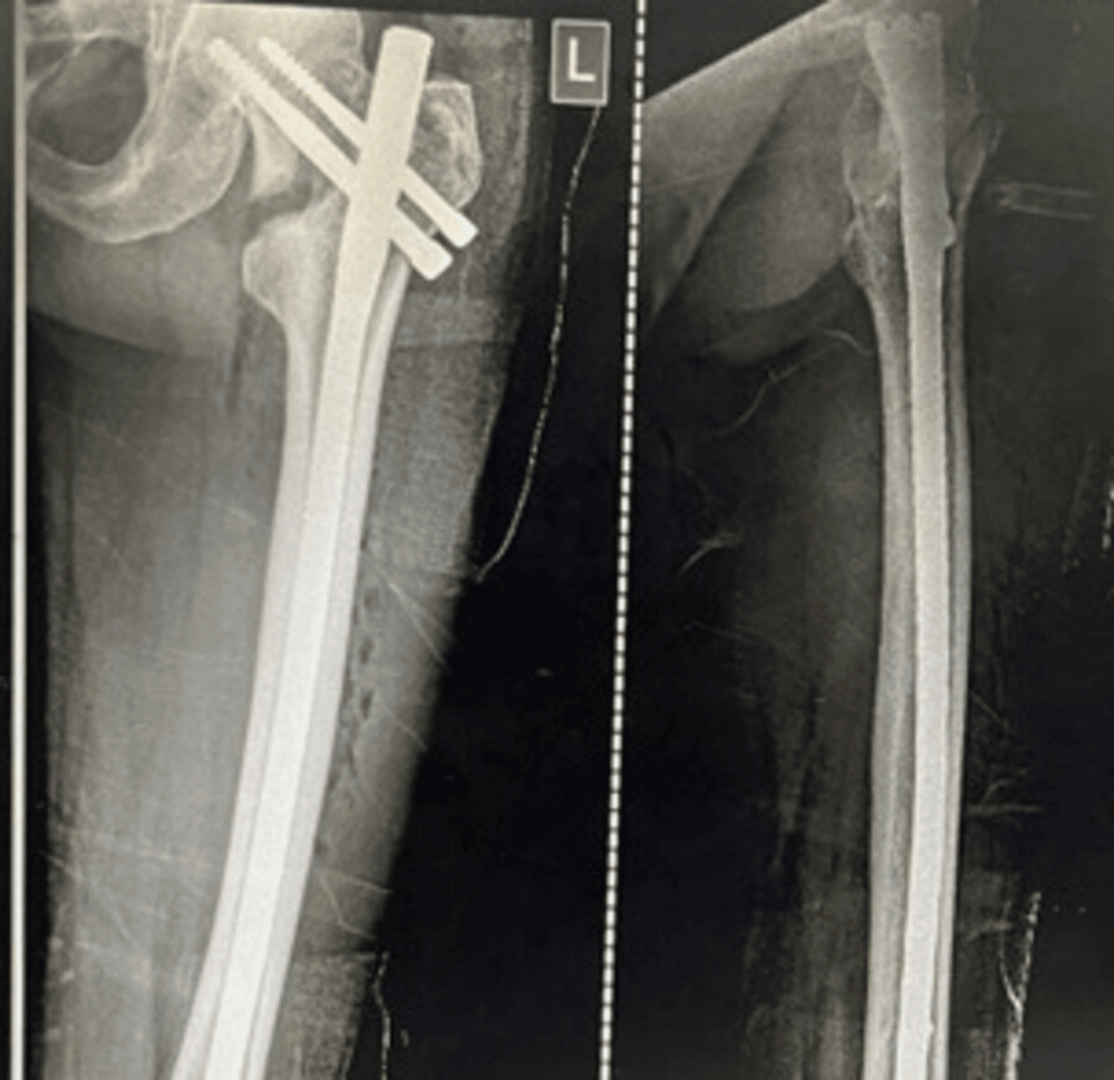
Immediate postoperative AP and lateral radiographs of the same patient treated by a long CMN The patient whose radiograph is shown in Figure 1 was treated with a long CMN. AP: anteroposterior, CMN: cephalomedullary nail.

**Figure 3:**
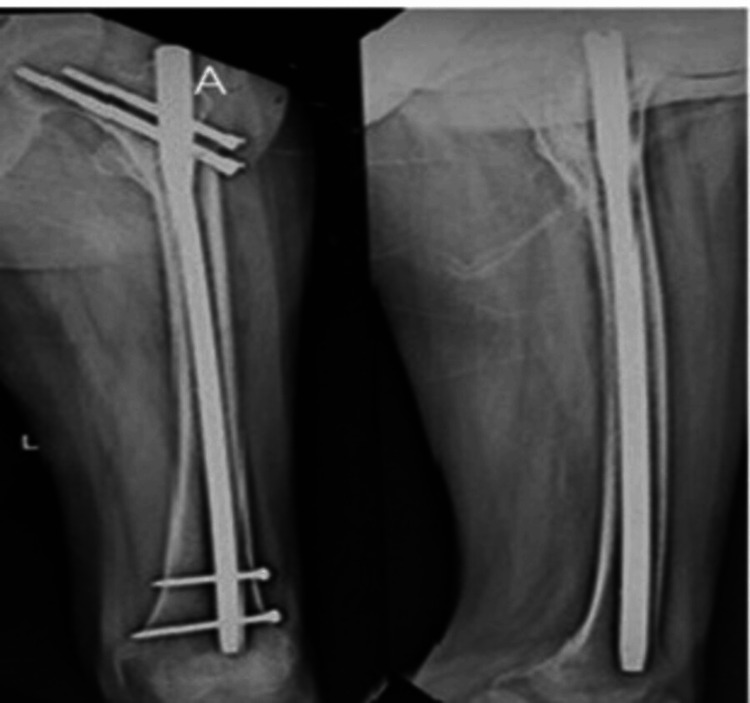
Six-month follow-up radiograph of the same patient The patient, whose radiograph is shown in Figure 1, underwent AP and lateral radiographic imaging of the left hip joint and femur six months postoperatively, showing radiological union of the intertrochanteric fracture. AP: anteroposterior.

Figures 1-3 display the preoperative and subsequent follow-up radiographs of a patient included in the study, diagnosed with an AO/OTA type 31A1 intertrochanteric fracture of the left femur. The patient was treated using a long CMN.

**Figure 4:**
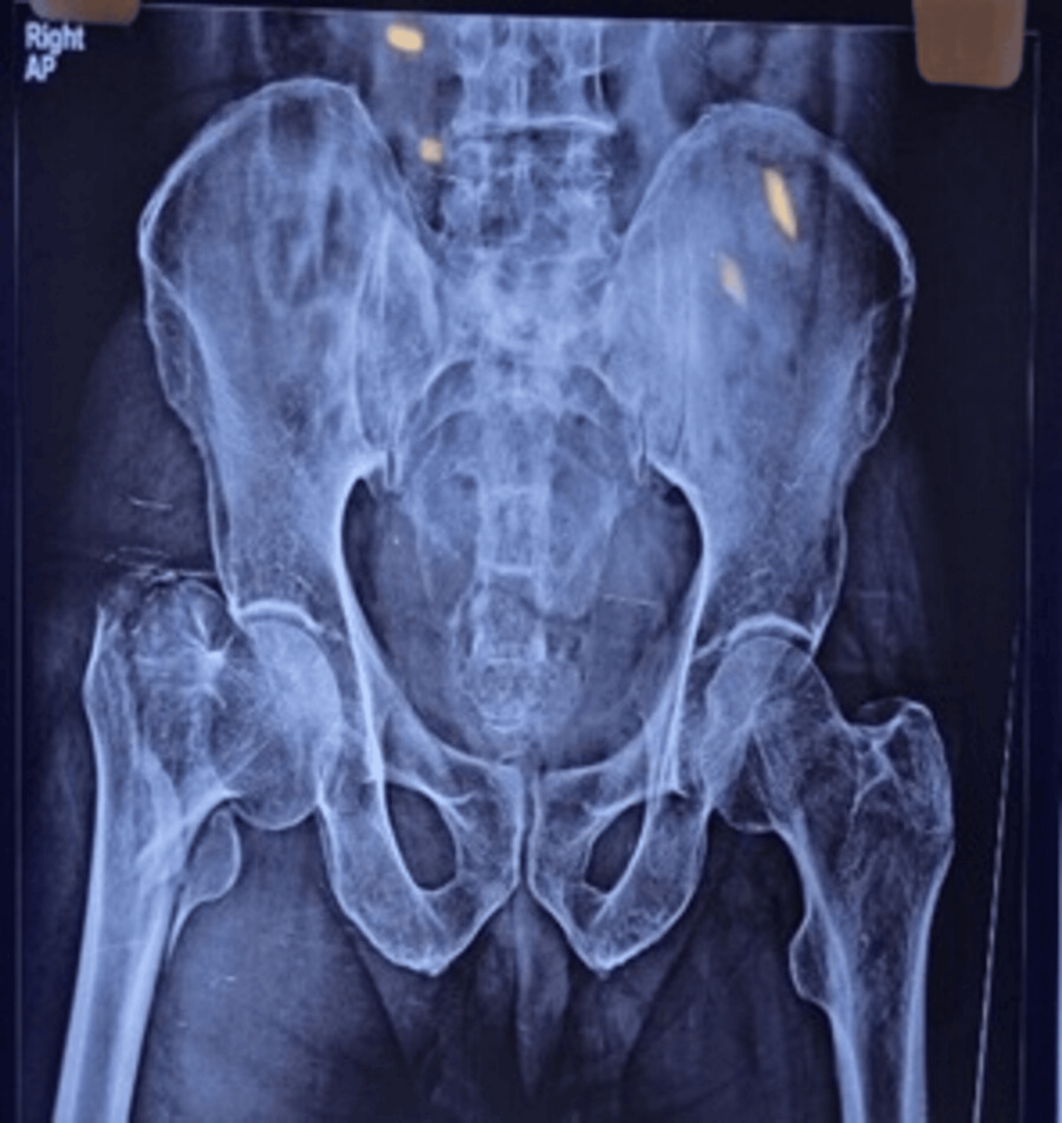
Preoperative AP radiograph of an adult patient showing right hip intertrochanteric fracture This is a preoperative radiograph, showing anteroposterior (AP) projection of the pelvis, bilateral hip joints, and proximal femurs, of a patient included in the study. The image reveals an AO/OTA type 31A2 intertrochanteric fracture. AO/OTA: Association for Osteosynthesis/Orthopaedic Trauma Association.

**Figure 5:**
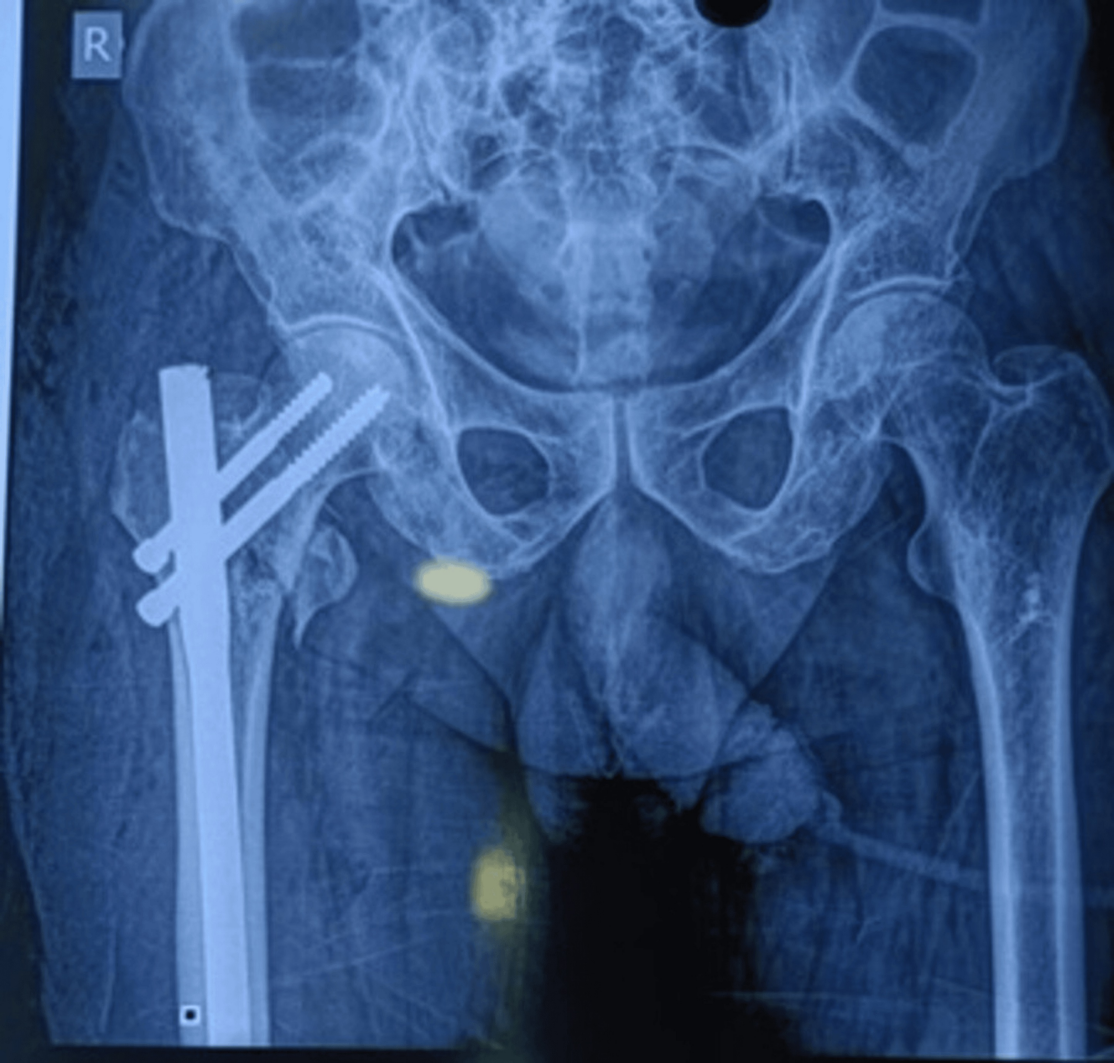
Immediate postoperative AP radiograph of the same patient treated by a short CMN The patient whose radiograph is shown in Figure 4 was treated with a short CMN. AP: anteroposterior. CMN: cephalomedullary nail.

**Figure 6:**
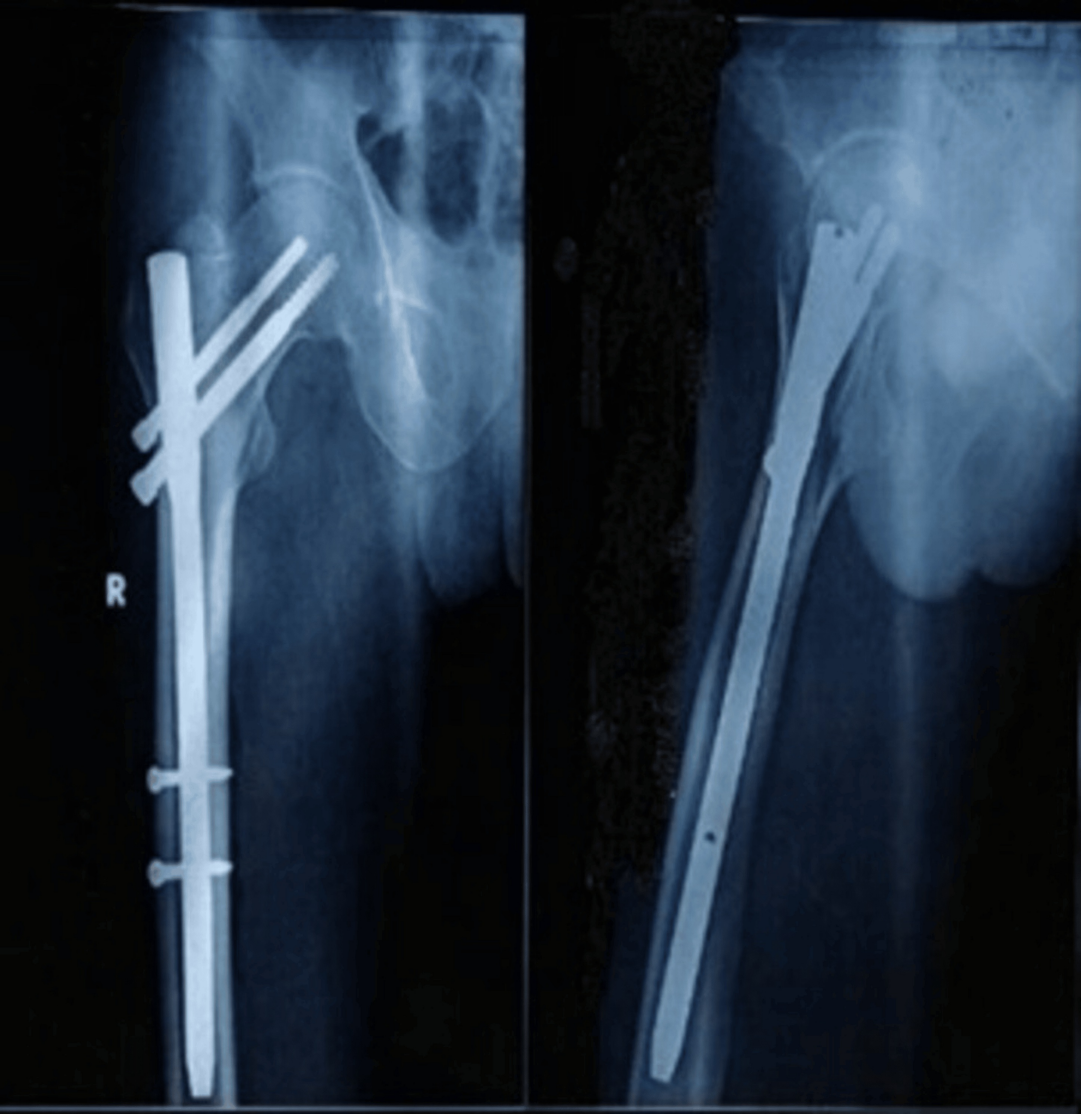
Eight-month follow-up radiograph of the same patient The patient, whose radiograph is shown in Figure 4, underwent AP and lateral radiographic imaging of the right hip joint and femur six months postoperatively, showing radiological union of the intertrochanteric fracture. AP: anteroposterior.

Figures 4-6 present the preoperative and follow-up radiographs of a patient included in the study, with an AO/OTA type 31A2 intertrochanteric fracture of the right femur, treated with a short CMN. 

## Discussion

The present study included a total of 50 subjects, with 25 each in the long and short CMN groups. The sample size of 50 patients was determined to balance practical considerations with the study’s objective of comparing functional outcomes between short and long CMNs in elderly patients with AO type 31A1 and 31A2 fractures. This number is consistent with prior orthopedic implant studies [12,13] and allows for reasonable statistical power (commonly 80%) to detect differences or equivalence in functional scores, complication rates, and operative parameters with moderate effect sizes. Recruitment and follow-up in this patient population can be challenging due to age-related comorbidities and mortality risk; therefore, a sample of 50 provides a feasible yet robust preliminary dataset. While larger, multicenter trials are recommended to validate these findings, this sample size supports meaningful comparative analysis and contributes valuable evidence for optimizing treatment strategies in this demographic.

Participants were categorized by age. In the long CMN group, 68% were aged 60-70 years, compared to 56% in the short CMN group. Additionally, 28% of subjects in both groups were between 71 and 80 years of age. A further 4% in the long CMN group and 16% in the short CMN group were above 80 years. Thus, the majority of patients in this study were aged between 60 and 70 years. However, no statistically significant difference was observed between the groups. Similar age distributions were reported by other studies: Temiz et al. [14] found most patients were aged 65-81 years, Berger-Groch et al. [15] reported a mean age of 81.2 ± 9.2 years, and Sharma et al. [12] observed an average age of 60.7 years. Nherera et al. [16] recorded a mean age of 77 years. According to Pfeilschifter et al. [17], bone mineral density progressively declines with age, increasing the risk of osteoporosis and, consequently, the incidence of fractures in the elderly. A statistically significant difference in intraoperative blood loss was observed between the groups. The long CMN group had a mean blood loss of 117.24 mL, compared to 94.84 mL in the short CMN group (p < 0.01). Similar findings were reported by Hou et al. [18], who concluded that long nails are associated with increased blood loss. Our results are also consistent with those of Schipper et al. [19], Duramaz and İlter [20], and Date et al. [21], all of whom found that short CMNs resulted in less blood loss. This may be attributed to the simplified insertion technique for short nails, which does not require prior drilling, thus minimizing cancellous bone loss and reducing bleeding. Thamyongkit et al. [22] reported no significant difference in blood transfusion rates between intermediate and long nails, although intraoperative blood loss was significantly higher with long nails. Literature comparing short and long nails has shown inconsistent results regarding hospital stay. Some studies reported longer hospitalization with long nails, while others found no significant difference [23]. In our study, the mean hospital stay was 10.04 days for the long CMN group and 10.60 days for the short CMN group, with the difference being statistically non-significant (p = 0.387). These findings are in line with those of Okcu et al. [13]. Postoperative hospital stay was also statistically non-significant (p = 0.521) between groups in our study. Similar results were reported by Singh et al. [24] and Rajnish et al. [25], where the mean duration of hospital stay ranged from 5 to 20.2 days in the short CMN group and 4.9 to 15.7 days in the long CMN group. This variability in length of stay among elderly patients with hip fractures may be influenced by factors such as postoperative rehabilitation protocols, institutional resources, and the need for extended nursing care. Overall, nail length appears to have no meaningful impact on hospital stay duration. The mean HHS was compared between groups at different postoperative intervals. On postoperative day 14, there was no statistically significant difference in HHS. However, by day 30, the mean HHS was 67.04 in the long CMN group and 69.92 in the short CMN group, with the difference being statistically significant (p = 0.02). At 3, 6, and 8 months postoperatively, the differences in mean HHS remained statistically significant (p = 0.000). Sharma et al. [12] reported a mean HHS of 75.37 between CMN and CMNA groups with no significant difference (p = 0.54), while Nherera et al. [16] and Loh et al. [26] also found no significant difference. Our findings diverge from these reports. Conversely, Dragosloveanu et al. [5] observed superior HHS outcomes in the short nail group, with a mean score of 84.76 ± 3.68, consistent with our findings and reaching statistical significance (p < 0.05). Regarding pain assessment using the VAS, no statistically significant differences were observed between groups at most intervals. This aligns with the findings of Dragosloveanu et al. [5], Nherera et al. [16], and Catania et al. [27]. However, at the 8-month follow-up, VAS scores were significantly different. Jha et al. [28] reported that most patients in both groups experienced only mild pain, with no cases of moderate or severe pain. Radiological union time was also evaluated. The mean time to union was 17.16 weeks in the long CMN group and 17.12 weeks in the short CMN group, a difference that was not statistically significant (p = 0.919). Hou et al. [18] also found no significant differences in union or complication rates between groups, indicating no advantage of long nails over short nails in terms of healing. There was no statistically significant difference in the number of cases of screw cutout or backout between the two groups. These findings are consistent with those of Dragosloveanu et al. [5], who observed two cases of cutout in the short CMN group and one case in the long CMN group. Screw cutout remains a common complication and often necessitates reoperation, frequently resulting from improper screw placement or increased tip-apex distance (TAD). Baumgaertner and Solberg [29] recommended maintaining a TAD of less than 25 mm to minimize the risk of screw cutout. Proper placement near the center of the femoral head in the lateral view further reduces torsional forces and the likelihood of failure [30].

This study has several limitations, including a small sample size, which may restrict the generalizability of the findings. The eight-month follow-up period, while sufficient for short-term evaluation, provides limited insight into the long-term survival of the implants. The study did not include unstable or reverse oblique intertrochanteric fractures, limiting its applicability to all fracture types. Surgeon-determined nail selection introduces potential selection bias. Additionally, the lack of blinding and randomization represents an important limitation, as these factors are critical for reducing bias. Future research should involve larger, multicenter studies with randomized and blinded designs to enhance the robustness and applicability of the results. Finally, since this study was conducted at a single center with a predominantly Central Indian population, its generalizability to other ethnic or geographic groups may be limited.

## Conclusions

This prospective study compared the outcomes between short and long cephalomedullary nailing in elderly patients with AO type 31A1 and 31A2 intertrochanteric femur fractures. The findings indicate that while both nail types are effective in achieving fracture union and restoring weight-bearing ability, short nails offer certain intraoperative advantages, such as reduced operative time and blood loss. Moreover, patients treated with short nails demonstrated better functional outcomes at several postoperative intervals, alongside comparable complication rates to those receiving long nails. Overall, these results suggest that short CMNs may offer a more advantageous option for elderly patients undergoing treatment for intertrochanteric femur fractures.
